# The Protective Effect of Broccoli Seed Extract against Lipopolysaccharide-Induced Acute Liver Injury via Gut Microbiota Modulation and Sulforaphane Production in Mice

**DOI:** 10.3390/foods12142786

**Published:** 2023-07-21

**Authors:** Bingyong Mao, Baojing Ren, Jiaying Wu, Xin Tang, Qiuxiang Zhang, Jianxin Zhao, Le Zhang, Wei Chen, Shumao Cui

**Affiliations:** 1 State Key Laboratory of Food Science and Resources, Jiangnan University, Wuxi 214122, China; maobingyong@jiangnan.edu.cn (B.M.); 15941665333@163.com (B.R.); wjyzz711@163.com (J.W.); xintang@jiangnan.edu.cn (X.T.); zhangqx@jiangnan.edu.cn (Q.Z.); zhaojianxin@jiangnan.edu.cn (J.Z.); chenwei66@jiangnan.edu.cn (W.C.); 2 School of Food Science and Technology, Jiangnan University, Wuxi 214122, China; 3 Department of Neonatology, Wuxi Children’s Hospital, Children’s Hospital Affiliated to Jiangnan University, Wuxi 214023, China; 4 National Engineering Research Center for Functional Food, Jiangnan University, Wuxi 214122, China

**Keywords:** broccoli seed extract, sulforaphane, intestinal microbiota, acute liver injury

## Abstract

Broccoli seed extract (BSE) is rich in glucoraphanin (GRP), which may be transformed by intestinal microbes into sulforaphane (SFN), a compound with strong anti-inflammatory and antioxidant activities. Liver injury usually presents with inflammation and oxidative damage. Thus, dietary BSE supplementation may be an effective approach for alleviating liver injury. In this study, a mouse lipopolysaccharide (LPS)-induced acute liver injury model was used to evaluate the preventive effect of BSE and explore the relevant mechanisms. Compared with the LPS model group, the mice in the BSE group showed significantly lower activities of aspartate aminotransferase (AST), alanine aminotransferase (ALT), alkaline phosphatase (ALP), and lactate dehydrogenase (LDH) and higher levels of catalase (CAT), superoxide dismutase (SOD) and glutathione peroxidase (GSH-Px) activity. Meanwhile, BSE significantly reduced the levels of pro-inflammatory cytokines (including IL-6 and TNF-α) in the liver and increased the level of anti-inflammatory factor (IL-10), indicating that BSE had a good preventive effect on acute liver injury. Additionally, after BSE intervention, the diversity of intestinal microbiota in the mice was higher than that in the LPS model group. The relative abundance of *Akkermansia* and *Lactobacillus* increased, while the relative abundance of *Xylanophilum* decreased. A correlation analysis revealed that the activities of SOD, GSH-Px, CAT and levels of IL-10 were positively correlated with the relative abundance of *Lactobacillus*. Furthermore, sulforaphane (SFN) and (Sulforaphane-N-Acetyl-Cysteine) SFN-NAC were detected in the urine of the mice after BSE intervention. Both q-PCR and an immunohistochemical analysis showed that BSE significantly regulated the expression level of the NF-κB (*IκB-α*, *NF-κB*) and Nrf2 (*Nrf2*, *p-Nrf2* and *HO-1*) signaling pathways in the liver. In conclusion, BSE was shown to reduce LPS-induced acute liver injury through the conversion of glucoraphanin into sulforaphane and the regulation of the gut microbiota composition. These results suggest that BSE could be a promising ingredient in functional foods.

## 1. Introduction

Liver is an important organ of the human body that is essential for the removal of toxins, secretion of endogenous and exogenous substances, and maintenance of metabolic homeostasis [1]. Many external factors, including food intake and serum micronutrient concentrations, participate in the pathogenesis of liver injury, the mechanism of which is complex and considered to be the result of multiple factors alone or in combination [2]. There are several types of liver injury, including alcohol-induced liver injury, drug-induced liver injury, infectious liver injury, and acute liver injury [3,4]. Among them, acute liver injury is a relatively common type and is mostly seen in patients without pre-existing liver disease [5]. Acute liver injury can be caused by lipopolysaccharides (LPS), which are endotoxins that act through Toll-like receptors present on the cell membrane surfaces of host cells. LPS enter the circulation and result in the stimulation of the immune system, the recruitment of immune cells to the liver, and the subsequent production of pro-inflammatory cytokines, which trigger an inflammatory reaction and result in acute liver injury [6]. The intraperitoneal injection of LPS induces acute liver injury in animals, which serves as a representative model of the disease that has been widely used to evaluate the pathogenesis of acute liver disease and drug intervention processes [7,8]. At present, some anti-inflammatory drugs are used to treat acute liver injury, but clinical data show that there are adverse effects of these medicines [9]. Therefore, the development of alternative treatments, such as the utilization of natural active substances in foods, to treat acute liver injury and inflammation can offer promising solutions.

As a main source of antioxidants, vegetables are important for human health. Broccoli (*Brassica oleracea var. italica Plenck*) is an annual plant in the family of Brassicaceae [10]. The People’s Republic of China was the largest producer of broccoli and cauliflower in 2019 [11]. Broccoli is rich in many nutrients, including minerals, vitamins, dietary fiber, glucosinolates, and phenolic compounds. Broccoli contains compounds that have anti-inflammatory, anticancer, and antibacterial activities and can also regulate metabolic syndrome and protect neurons and the kidneys from damage [12,13]. In China, broccoli seed extract (BSE) was authorized as a novel food by the National Health Commission in 2017, and the recommended dietary intake does not exceed 1.8 g daily. According to the product standard, the content of glucoraphanin (GRP) in broccoli seed extract (BSE) is 13~20% (*w*/*w*). The GRP in broccoli can be readily converted into highly bioactive sulforaphane (SFN) and other beneficial metabolites by myrosinase or intestinal bacteria, further increasing its beneficial biological activity [13]. Animal and clinical studies have shown that SFN has a variety of health benefits, including strong antioxidant activity, the activation of the anti-inflammatory Nrf2 signaling pathway, and the alleviation of neuroinflammation and biochemical abnormalities related to autism [14]. In addition, SFN inhibits two major cytochrome P450 enzymes (3A4/5 and 2D6) in human liver microsomes, which participate in various important reactions in human drug metabolism [15]. These results suggest that broccoli seed extract is a promising dietary source of anti-inflammatory compounds that could serve as an excellent treatment for acute liver damage.

In this study, we evaluated the intervention effect of broccoli seed extract on LPS-induced acute liver injury in mice and analyzed its related mechanism. The degree of acute liver injury was evaluated through the histopathological assessment of the liver, combined with the determination of serum antioxidant-related enzyme activity and the liver inflammatory factor level. The changes in intestinal microbiota composition were then analyzed, and the correlations between the intestinal microbiota and physiological and biochemical indicators were determined through network visualization. The transcription and expression levels of key signal pathways were analyzed via q-PCR and immunohistochemistry. The results of this study serve as an important reference for the use of bioactive substances to treat acute liver injury and inflammation and will help to progress the development of functional foods in the future.

## 2. Materials and Methods

LPS was provided by Sigma-Aldrich (Saint Louis, MO, USA). BSE containing 20% GRP was purchased from Pioneer Herb Industrial Co. Ltd. (Ganzhou, China). The enzyme-linked immunosorbent test (ELISA) kits were obtained from Enzyme-linked Biotech Co., Ltd. (Shanghai, China). All analytical-grade chemicals were supplied by Sigma (Shanghai, China).

### 2.1. Animal Experimental Design

Twenty-four male SPF C57BL/6J mice (18 ± 2 g, 6 weeks of age) were obtained from Shanghai Animal Research Center and housed in a standard environment. The temperature was controlled at 21–25 °C, and the humidity was controlled between 40% and 60%. A 12 h light/dark cycle was used. The mice had free access to commercial diets (Synergy Bio Pvt. Ltd., Nanjing, China) and sterile water. The commercial diets mainly consisted of corn, wheat, imported fish meal, chicken meal, soybean meal, soybean oil, amino acids, vitamins, and minerals.

After 7 days of adaptive feeding, the mice were randomly divided into 3 groups (*n* = 8 for each group), namely, the control group, the model group, and the broccoli seed extract (BSE) group. The model of acute liver injury was established through LPS intervention. The intervention period lasted for 14 days. For the control group, the mice were gavaged daily with 0.2 mL of skimmed milk (13%, *w*/*v*). For the model group, the mice were gavaged daily with 0.2 mL of skimmed milk (13%, *w*/*v*). For the BSE group, the mice were gavaged with 0.2 mL of BSE (370 mg kg^−1^ day^−1^) dissolved in 0.2 mL of skimmed milk (13%, *w*/*v*). The body weight of the mice was recorded every two days. The food and water intakes were recorded daily. Mice feces were collected after 14 days of intervention.

On day 15, the mice in the model group and BSE group were intraperitoneally injected with bacterial lipopolysaccharide (LPS) (6 mg/kg body weight) [16], while the mice in the control group were intraperitoneally injected with NaCl solution (0.9%, *w*/*v*). Then, 4 h after injection, the mice were subjected to inhalation anesthesia with isoflurane, with an oxygen flow rate of 2 L/min and an isoflurane anesthesia concentration of 4%. After anesthesia, blood was collected from the orbit, and the mice were sacrificed. Then, a small incision was made with scissors on the left side of the midline of the abdomen up to the point of sternal protrusion, followed by a transverse incision along the posterior edge of the ribs to expose the abdominal cavity. The liver and spleen tissues were collected, and part of the liver tissue was fixed in paraformaldehyde (4%, *w*/*v*).

All the animal experiment procedures were approved by the Ethics Committee of Jiangnan University (JN.NO20201115c0701230[309]).

### 2.2. Measurement of Anti-Inflammatory Biomarkers in Serum

A Mindray BS-180 blood chemistry analyzer (Shenzhen Mindray Bio-Medical Electronics Co., Ltd., Shenzhen, China) was used to determine the total aspartate aminotransferase (AST), alanine transaminase (ALT), alkaline phosphatase (ALP), lactate dehydrogenase (LDH), total cholesterol (TC), triglycerides (TG), and C-reactive protein (CRP) levels in the serum. The determination was performed according to the manual of the analyzer and the reagents. All the levels of the serum indicators were in the detection ranges of the corresponding parameters. All the standard reagents were obtained from Mindray (Shenzhen Mindray Bio-Medical Electronics Co., Ltd., Shenzhen, China).

### 2.3. Assessment of the Anti-Inflammatory and Antioxidant System in Hepatic Tissues

The partial livers were homogenized with 0.9% (*w*/*v*) normal saline and centrifuged at 14,000× *g* for 10 min at 4 °C, and we retained the supernatant for detection. The levels of the hepatic tissue inflammatory factors IL-6, TNF-α, and IL-10 were analyzed with ELISA kits (R&D Systems Co., Ltd., Minneapolis, MN, USA.). The levels of oxidative stress biomarkers in the liver, including catalase (CAT), superoxide dismutase (SOD), glutathione peroxidase (GSH-PX), and malondialdehyde (MDA), were analyzed using commercial kits (Jiancheng Biotechnology Co., Ltd., Nanjing, China). All the determinations were performed strictly following the instructions and manuals of the corresponding kits.

### 2.4. Liver Histopathology

Liver histopathology was observed according to the research of Markiewicz-Kijewska et al. [17], with minor modifications. Samples of the liver tissues were injected into paraffin after being treated in 4% formalin. Both hematoxylin and eosin were used to stain the 4 µm paraffin sections (H&E), which were then observed with a fluorescence microscope after staining.

### 2.5. Intestinal Microbiota Analysis

Total DNA was obtained from the cecal sample using a commercial kit (MoBio, Carlsbad, CA, USA). Then, the 16S rRNA (V3–V4) was amplified using 338F/806R primers. The PCR products were purified with a commercial reagent (Qiagen GmbH, Hilden, Germany) and then used to create DNA libraries. The libraries were sequenced on the Illumina MiSeq platform at Jiangnan University (Wuxi, China). The sequence information was divided into operational taxonomic units with 97% similarity. SIMCA (v 14.1) was used to analyze the overall gut microbial structure using hierarchical clustering plots and principal coordinates analysis (PCA) scores. The distribution of the gut microbial genera was revealed using STAMP (v 2.1.3). R software (v4.0.3) and Cytoscape were used to visualize the connection between the proportions of gut microbial genera and inflammation.

### 2.6. Quantitative RT-PCR

A StepOnePlus RT-PCR System (AB, CA, USA) and commercial q-PCR kits (Takara, Dalian, China) were used for the extraction and reverse transcription of the total hepatic RNA. The primer sequences used for RT-PCR are listed in Table 1. The expression of the targeted genes was estimated using 2^−ΔΔct^.

### 2.7. Immunohistochemical Analysis

The formalin-fixed, paraffin-embedded liver tissues were sectioned at 5 μm. Then, the sections were dewaxed in xylene and rehydrated through a graded series of alcohol to water. The liver slices were first treated for 60 min with primary antibodies against Iκ-Bα, NF-κB, Nrf2, p-Nrf2, and HO-1 (obtained from Abcam, Cambridge, UK) and then incubated for 120 min with biotinylated anti-mouse secondary antibodies (obtained from Abcam, Cambridge, England). Finally, the tissue sections were then incubated with streptavidin-horseradish peroxidase (Abcam, Cambridge, UK), and a light microscope was used to measure the brown shade under 3,3’-diaminobenzidine tetrahydrochloride [18]. Three randomly selected areas were observed per slide, and the mean density was calculated using ImageJ software.

### 2.8. Statistical Analysis

The results were expressed as the mean ± SEM. The data were analyzed using GraphPad Prism (v 7.0) (Informer Technologies, Inc., CA, USA). According to Tukey’s tests, the differences between the three groups were analyzed using one-way ANOVA, and *p* < 0.05 was considered statistically significant.

## 3. Results

### 3.1. Effects of Broccoli Seed Extract on Body Weight and Food and Water Intakes of Mice

**Figure 1:**
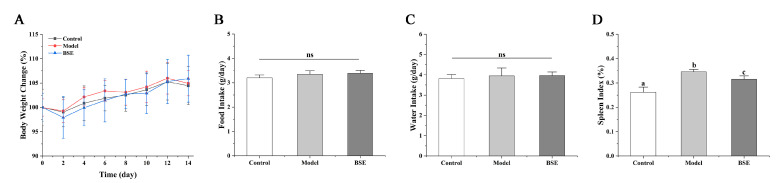
Changes in body weight (**A**), food intake (**B**), water intake (**C**), and splenic index (**D**) in mice of different groups. a, b, c: different letters indicate significant differences. ns, not significant.

The changes in the body weight and the food and water intakes of the three groups of mice were determined on days 0, 2, 4, 6, 8, 10, 12, and 14, and there were no significant differences between the three groups (Figure 1A–C). The splenic index is a ratio of the weight of the spleen to the body weight of mice, and the determination of the “splenic index” can help one to judge whether the model has been successfully established, because LPS can cause splenomegaly. The results showed that the splenic index of the model group was significantly higher than that of the control group (*p* < 0.05), indicating that LPS treatment may have caused splenomegaly in the mice of the model group (Figure 1D). BSE intervention may help to inhibit the splenomegaly of mice.

### 3.2. Pretreatment with Broccoli Seed Extract Improved Liver Function of Liver Injury Mice

**Figure 2:**
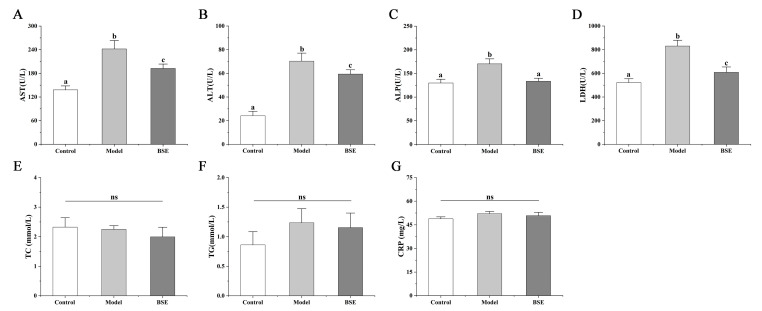
Changes in serum AST (**A**), ALT (**B**), ALP (**C**), LDH (**D**), TC (**E**), TG (**F**), and CRP (**G**) concentrations in mice of different groups. a, b, c: different letters indicate significant differences. ns, not significant.

Aspartate aminotransferase (AST), alanine aminotransferase (ALT), alkaline phosphatase (ALP), and lactate dehydrogenase (LDH) are serum biomarkers of liver injury. The AST, ALT, ALP, and LDH levels in the mice of model group were significantly higher than those of the control group (Figure 2A–D). BSE treatment could significantly reduce the serum levels of AST, ALT, ALP, and LDH compared to the model group. The levels of total cholesterol (TC), triglycerides (TG), and C-reactive protein (CRP) were not significantly different between the three groups (Figure 2E–G).

### 3.3. Pretreatment with Broccoli Seed Extract Prevented the Inflammatory Response and Increased Antioxidant Ability in Liver Injury Mice

**Figure 3:**
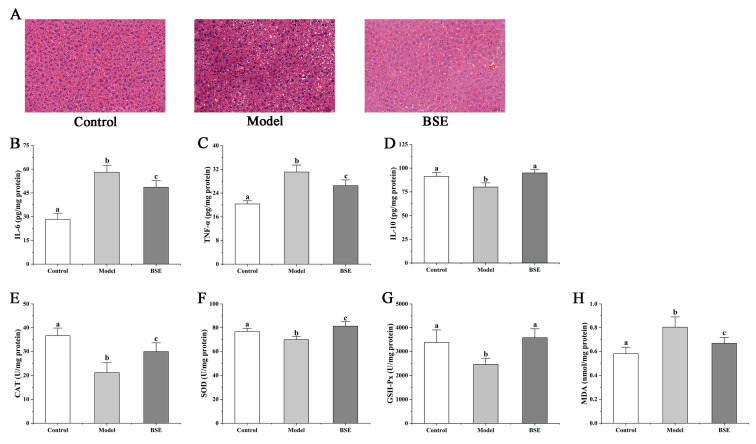
Histologic sections of the liver stained with H&E (**A**) and changes in hepatic IL-6 (**B**), TNF-α (**C**), IL-10 (**D**), CAT (**E**), SOD (**F**), GSH-PX (**G**), and MDA (**H**) levels in mice of different groups. a, b, c: different letters indicate significant differences.

The liver tissue slices, inflammatory factors and relevant biochemical indexes reflect the effect of BSE treatment on liver injury. Compared with the control group, the destruction of the liver tissue structure, cellular cavities, and cell infiltration after LPS treatment were observed in the model group (Figure 3A). However, there were fewer adverse conditions in the BSE group than in the model group, suggesting that BSE intervention helped to reduce the liver damage caused by LPS. Furthermore, the levels of liver pro-inflammatory factors IL-6 and TNF-α were significantly higher in the model and BSE groups than in the control group, while the level of anti-inflammatory factor IL-10 showed the opposite trend. These results show that increased inflammation liver injury can be alleviated through elevated anti-inflammatory cytokines levels resulting from BSE pretreatment (Figure 3B–D).

Pretreatment with BSE significantly increased the antioxidant capacities, as indicated by the elevated levels of catalase (CAT), superoxide dismutase (SOD), and glutathione peroxidase (GSH-Px) in the liver of the mice in the BSE group compared to those in the model group, while malondialdehyde (MDA) was lower in the BSE group than in the model group (Figure 3E–H).

### 3.4. Pretreatment with Broccoli Seed Extract Regulated the Mice Intestinal Microbiota Structure

**Figure 4:**
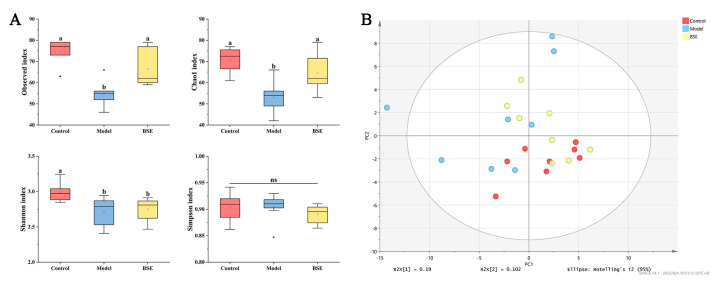
Abundance and diversity of intestinal microbiota (**A**) and principal coordinate analysis (PCoA) of intestinal microbiota (**B**) in the mice of three different groups. a, b: different letters indicate significant differences. ns, not significant.

In this study, 16S rRNA high-throughput sequencing technology, combined with principal component analysis (PCA), was used to reveal the characteristics of the intestinal microbiota and their relationships in the three groups. The model group had the lowest microbial diversity, while the BSE group also showed decreased microbial diversity compared to the control group (Figure 4A). In terms of richness, there was no significant difference between the model group and BSE group, and both were lower in richness than the control group (Figure 4A). Furthermore, there was no significant difference between the BSE group and control group in intestinal microbiota composition, whereas a significant difference was observed between the model group and the other two groups (Figure 4B).

**Figure 5:**
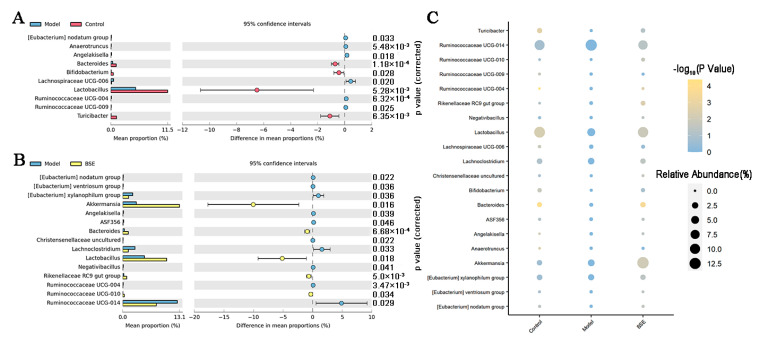
Intestinal microbiota in model vs. control (**A**), model vs. BSE (**B**), and all three groups (**C**) compared on the genus level.

In addition, the relative abundance of specific bacterial species in the three groups differed. Compared with the control group, the relative abundance of *Bacteroides*, *Bifidobacterium*, *Lactobacillus*, and *Turicibacter* was significantly decreased and the relative abundance of *Lachnospiraceae UCG-006* was significantly increased in the model group. There was a significant increase in the relative abundance of *Akkermansia* in the BSE group as compared to the control and model groups (Figure 5).

### 3.5. Relationships between Intestinal Microbiota and Biochemical Indicators in Serum and Liver and Inflammatory Factors in Liver and Urine Nutrients

**Figure 6:**
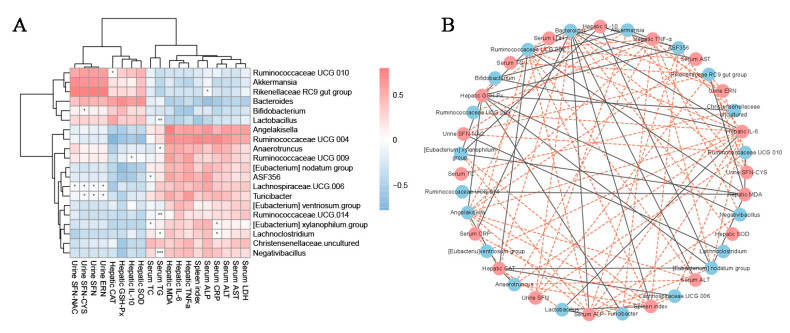
The relationship between the key intestinal microbial phylotypes and the parameters related to acute liver injury and inflammation. Heatmap of Spearman’s correlations, where the intensity of the color represents the degree of association (**A**). Correlation network built between bacterial species and microbial metabolic pathways, where the edge width and color (red: positive; blue: negative) are proportional to the correlation strength (**B**). The significant correlations between key intestinal microbial phylotypes (horizontal) and key indicators (vertical) are indicated as * (*p* < 0.05), ** (*p* < 0.01) and *** (*p* < 0.001).

Specific microbial species are correlated with certain biomarkers in both the serum and liver, as well as inflammatory factors in the liver and nutrient levels in urine. In the liver, SOD, IL-10, GSH-Px, and CAT had the greatest positive correlations with Bacteroides, Bifidobacterium, and Lactobacillus and a higher negative correlation with *Ruminococcaceae UCG 004*, nodatum, ASF356, and *Lachnospiraceae UCG 006*. In the liver, IL-6, TNF-α, and MDA had the most positive correlations with Angelakisella and *Ruminococcaceae UCG 004* (Figure 6A,B) and more negative correlations with *Bacteroides*, *Bifidobacterium* and *Lactobacillus*. As for LDH, AST, ALT, CRP, ALP, TG and TC in the serum, the positive correlations with *Angelakisella* and *Ruminococcaceae UCG 004* were the highest, and the negative correlations with Bacteroides, Bifidobacterium and *Lactobacillus* were the highest (Figure 6A,B).

Erucin (ERN), sulforaphanin (SFN), sulforaphane-cysteine (SFN-CYS), and Sulforaphane-N-Acetyl-Cysteine (SFN-NAC) were only detected in the urine of mice in the BSE group (Table 2 and Table 3), indicating that these substances may be generated from the transformation of glucosinolate present in BSE by intestinal bacteria. ERN, SFN, SFN-CYS, and SFN-NAC had the highest positive correlations with the *Ruminococcaceae UCG 010*, *Akkermansia*, and *Rikenellaceae RC9* gut groups and the highest negative correlation with *Lachnoclostridium*.

### 3.6. Influence of Broccoli Seed Extract on Hepatic mRNA Levels Associated with Inflammation and Antioxidation in Liver Injury Mice

**Figure 7:**
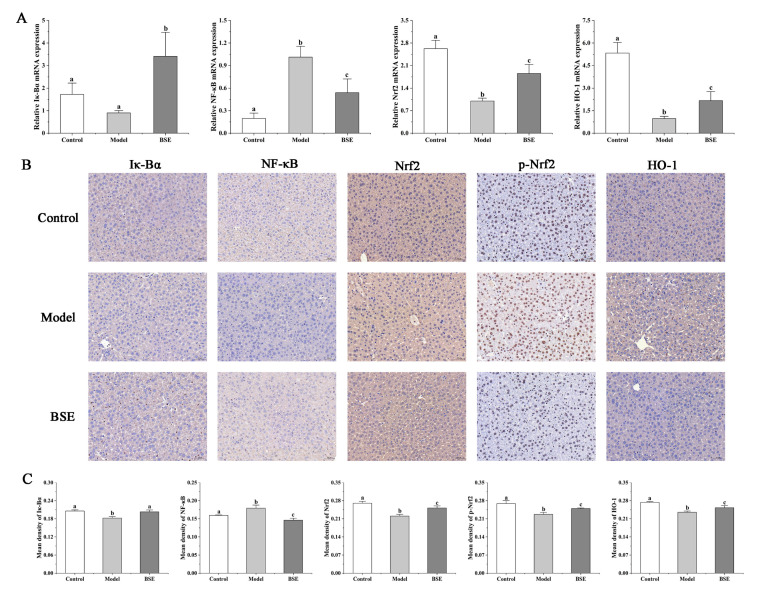
Influences of BSE in regulating the Nrf2 and NF-κB signal pathways. (**A**) Relative mRNA expression levels of Iκ-Bα, NF-κB, Nrf2, and HO-1; immunohistochemical staining (**B**) and mean density (**C**) of Iκ-Bα, NF-κB, Nrf2, p-Nrf2, and HO-1. a, b, c: different letters indicate significant differences.

The expression levels of mRNA related to acute liver injury and inflammation were determined to further evaluate the effects of BSE on LPS-treated mice. The mRNA levels of IκB-α and NF-κB inflammatory factors were quantified and compared between the groups. The mRNA levels of IκB-α in the BSE group were higher than that in the model group (Figure 7A), whereas NF-κB mRNA levels were lower. Furthermore, the average density of IκB-α in the BSE group was lower than that in the model group, while the average density of NF-κB was higher than that in the model group (Figure 7B,C). The levels of Nrf2, p-Nrf2, and HO-1 in the model group and BSE group were lower than those in the control group, which were higher in the BSE group compared to the model group (Figure 7A,C).

## 4. Discussion

Liver injury is mainly related to ROS production and oxidative stress. It also increases the levels of acute phase protein, C-reactive protein (CRP), and other relevant inflammatory factors in the liver and serum [19]. It is reported that some active plant polysaccharides help to alleviate liver injury by reducing the level of inflammatory factors. For example, Schisandra chinensis acidic polysaccharide (SCAP) significantly improved ethanol-induced liver injury and the levels of AST and ALT in HepG2 cells, as well as the level of TG in liver tissue [20]. Jasmine polysaccharide (GPS) inhibited Toll-like receptor 4 (TLR4)/nuclear factor kappa-B (NF-κB) signaling, which can reduce the expression of inflammatory factor genes and improve liver inflammation [21].

Broccoli is rich in a variety of active substances, especially GRP, which can be metabolized into more bioactive SNF. Broccoli flower bud extract has been shown to reduce alcohol-induced oxidative stress in C57BL/6 mice and improve alcohol-induced liver injury [22], which may be related to its SNF content. In our study, BSE intervention decreased the levels of pro-inflammatory cytokines IL-6 and TNF-α, increased the levels of anti-inflammatory cytokine IL-10, as well as the levels of related antioxidant biomarkers (CAT, SOD, GSH-Px), decreased MDA, and significantly decreased LPS-induced liver injury. These results are in agreement with a population study that used sulforaphane-cysteine and SFN-NAC as broccoli uptake markers and concluded that the decrease in the inflammatory factor level was closely related to the increase in the number of broccoli-related metabolites [23]. It is well-known that SFN is an effective activator of Nrf2, which dissociates Nrf2 from Keapl and translocates it to the nucleus. Nrf2 can inhibit inflammation through redox control and can also inhibit the up-regulation of the transcription of pro-inflammatory cytokine genes related to the NF-κB signaling pathway [24]. At the same time, HO-1 is the target gene of Nrf2, and the increase in Nrf2 can boost HO-1 activity, thus inhibiting NF-κB-mediated transcription [25]. This study also presented a pathway analysis showing that BSE activated Nrf2, inhibited the NF-κB signaling pathways, and increased HO-1 significantly. After LPS treatment, the liver tissue structure and cell cavity, were significantly damaged, with cell infiltration being observed, and the BSE treatment had a good improvement effect. These results indicated that supplemental BSE alleviates LPS-induced liver injury and is related to the production of sulforaphane (SFN).

If the GPR present in BSE can be converted into SFN, it will have higher biological activities. However, SFN is unstable and volatile. Thus, the main existing form of SFN in natural plants is its inert precursor, GRP [26]. The questions of whether GRP is transformed in the intestine and how efficient this conversion is are related to the microbial composition in the intestine of the host [27]. In this study, the dominant bacteria of the intestinal microorganisms in mice were *Lactobacillus*, *Bacteroides*, and *Turicibacter*. *Ruminococcaceae UCG-014*, *Akkermansia*, and *Lactobacillus* became the dominant intestinal genera after LPS treatment. However, humans and mice significantly differ in the composition of the intestinal microbiota, which also varies between individuals. Therefore, the ability and efficiency of human intestinal microbes in converting GRP into SFN will vary [28]. In our previous study, we found that *Lactobacillus plantarum*, *L. paracasei*, *B. longum*, and *B. pseudocatenulatum* can metabolize GRP, and *B. longum* isolated from human feces can convert GRP into sulforaphane [29]. Thus, the simultaneous intake of BSE (rich in GRP) and *B. longum* can promote the production of SFN in the human intestine, especially for people without the ability to metabolize GPR into SFN, which will further mitigate liver injury and other inflammatory diseases. In this study, we found that BSE can reduce liver injury by transforming GRP into sulforaphane in mice, but the dose effects of BSE in this amelioration were not revealed. Although the minimum effective dose of SFN for oral administration has been reported to be 3–10 μmol/kg in mice [30], it is still necessary to know the dose–effect relationships between SFN and its anti-inflammatory activities in the intestine. In this way, such information can be used as a guide for our dietary intake of Broccoli or BSE based on the GPR content to ensure that the body produces enough SFN to exert anti-inflammatory effects.

## 5. Conclusions

In summary, GRP, which is found at a high concentration in BSE, is converted into SFN by intestinal microbes, which ameliorates LPS-induced acute liver injury by activating Nrf2 and inhibiting the NF-κB signal pathway. BSE supplementation also altered the intestinal microbiota composition, which had strong correlations with GRP and SNF metabolism. This study provides a new strategy for the use of edible phytochemicals to alleviate liver injury. Broccoli seed extract (BSE) and other vegetables rich in GRP are valuable food resources and will advance the research and development of functional foods.

## Figures and Tables

**Table 1:** Gene sequences of primers used in this study.

Gene	Forward Primer (5′-3′)	Reverse Primer (5′-3′)
*Iκ-Bα*	5′-ACCAACCAGCCAGAAATCG-3′	5′-TCACAGGCAAGGTGTAGAGGG-3′
*NF-κB*	5′-CGCCCCTTATCGACCACC-3′	5′-CCTTCTCCCAAGAGTCGTCCA-3′
*Nrf2*	5′-CCTCCGCTGCCATCAGTCAGT-3′	5′-TCGGCTGGGACTCGTGTTCA-3′
*HO-1*	5′-ACAGAAGAGGCTAAGACCG-3′	5′-CAGCCCTACTTGGTTAGAAT-3′
*GADPH*	5′-AGGTCGGTGTGAACGGATTTG-3′	5′-GGGGTCGTTGATGGCAACA-3′

**Table 2:** Identification and chemical properties of metabolites detected in the urine of mice in the three groups.

Name	Molecular Formula	Retention Time (min)	Mass	MS Spectral Data *m*/*z*
SFN	C_6_H_11_NOS_2_	9.59	177.28	159, 155, 142, 114, 110
SFN-CYS	C_9_H_18_N_2_O_3_S_5_	3.15	298.45	299, 183, 159, 142, 130, 114
SFN-NAC	C_11_H_20_N_2_O_4_S_3_	7.93	340.48	341, 183, 155, 142, 118, 114
ERN	C_6_H_11_NS_2_	14.00	161.29	155, 142, 118, 114, 110

**Table 3:** Concentration of SFN and other metabolites in the urine of mice in the three groups.

Group	SFN (μM)	SFN-CYS (μM)	SFN-NAC (μM)	ERN (μM)
Control	-	-	-	-
Model	-	-	-	-
BSE	55.77 ± 5.22	3.01 ± 0.44	53.75 ± 5.46	1.39 ± 0.08

“-”: not detected.

## Data Availability

Data are contained within the article.
